# Assessment of reproductive challenges and nutritional practices on Pennsylvania sow farms

**DOI:** 10.1093/tas/txaf139

**Published:** 2025-10-10

**Authors:** Samantha R Yankocy, Rachel E Walker, Elizabeth A Hines, Claire Stenhouse

**Affiliations:** Department of Animal Science, College of Agricultural Sciences, Pennsylvania State University, University Park, Pennsylvania, USA; Department of Nutritional Sciences, College of Health and Human Development, Pennsylvania State University, University Park, Pennsylvania, USA; Department of Animal Science, College of Agricultural Sciences, Pennsylvania State University, University Park, Pennsylvania, USA; Department of Animal Science, College of Agricultural Sciences, Pennsylvania State University, University Park, Pennsylvania, USA

**Keywords:** health, reproduction, retinol, swine, vitamin A, vitamin D

## Abstract

Pennsylvania is ranked 12^th^ in the United States of America in pork production and hosts a diverse population of farms of different herd sizes, management techniques, and reproductive issues. Despite the appreciation of this diversity, these differences have not been systematically quantified. Variations in housing systems, feeding strategies, and overall management can influence reproductive outcomes and animal health. Furthermore, significant knowledge gaps persist regarding the vitamin A and D requirements of modern sows necessary to support optimal health and reproductive function. Establishing baseline serum concentrations of these vitamins is essential for defining nutritional adequacy in contemporary swine production systems. A survey was distributed to Pennsylvania pork producers assessing herd health, reproductive challenges, and management strategies, aiming to characterize statewide variation. A total of 45 responses that met the inclusion criteria were analyzed. Respondents were also asked about their willingness to participate in a follow-up blood sampling effort to evaluate serum vitamin A and D levels; four respondents agreed to participate. Survey responses were analyzed using frequency statistics, and serum data were evaluated using parametric and non-parametric statistics. Differences were observed when comparing health issues, feed source, veterinary records, and mortality between herd sizes, and reproductive issues by frequency of selection (*P *< 0.05). Serum retinol concentrations differed by farm (*P *< 0.10), with sows from Farm 1 having higher values than Farm 2 (*P *< 0.05). Serum retinol concentrations were not affected by parity or pregnancy status (*P *> 0.05). Serum 25(OH)D concentrations were not affected by parity but varied by farm, being greater in Farm 2 than Farm 3 (*P *= 0.01). These data indicate that Pennsylvania swine producers face diverse health and reproductive challenges, influenced in part by herd size and associated management strategies. As such, education and management strategies to optimize herd nutrition, health, and reproduction should account for these contextual differences.

## Introduction

Within the United States, Pennsylvania has the 12^th^ largest pig inventory (USDA 2025). In Pennsylvania, 91% of swine farms have fewer than 2,000 animals, collectively accounting for 12% of the state’s pig population (Cook and Schulz 2023). Many of these small farms classify themselves as “backyard farms”, composed of less than 25 breeding animals, with an emphasis on purebred genetics and market show hogs. However, most of the swine found in Pennsylvania are from large (2000+ head) farms that are both independent and corporate owned. This diversity of swine management styles highlights the necessity of ensuring both small and large producers have access to relevant nutrition, reproduction, and management information to maximize animal health and productivity.

Optimal nutrition is fundamental to animal health, growth, and productivity. Vitamins are an critical component of the diet, essential not only for overall animal health but also for optimal reproductive performance, influencing fertility, embryonic development, and pregnancy outcomes across mammalian species. Current recommendations for gestating and lactating sows are 8,398 and 11,932 IU/day of vitamin A and 1,680 and 4,773 IU/day of vitamin D, respectively (NRC 2012). Evidence indicates that nutritional intake of both vitamins A and D is closely linked to reproductive outcomes in sows. Restricted vitamin A intake increases embryonic mortality and stillbirths, whereas supplementation above NRC levels reduces the number of stillborn piglets (Brief and Chew 1985; Coffey and Britt 1993; Lindemann et al. 2008). Similarly, vitamin D supplementation improves piglet muscle development and birth weight, even when supplemented to sows without clinical deficiency (Coffey et al. 2012; Hines et al. 2013).

Beyond reproductive and growth performance, vitamins A and D are important regulators of overall health. Vitamin A deficiency is associated with xerophthalmia (Ross and Harrison 2007), impaired immune responses and increased susceptibility to infection (Surman et al. 2020). Vitamin D deficiency disrupts calcium and phosphate homeostasis, leading to impaired musculoskeletal development and function (DeLuca 2014). Poor reproductive performance often reflects broader herd health or nutritional challenges, underscoring the importance of appropriate nutritional management.

Sows today produce more piglets per litter than ever before, with an average litter size of 11.65 in 2024 in the United States (USDA 2025). Given this striking change in sow physiology, it could be speculated that this significant improvement in productivity may have led to alterations in the nutritional requirements for gestating and lactating sows; a concept which warrants further investigation. Importantly, there is limited up-to-date information on the recommended serum concentrations of important nutrients, such as vitamins A and D, in the modern sow. It is believed that the National Research Council (NRC) recommendations are not sufficient to meet the vitamin requirements of the modern sow (Darroch 2001). However, there is often debate on the interpretation of whether a required amount of a vitamin is simply that which prevents clinical symptoms of deficiency or whether it is the amount that maximizes the performance of an animal. Understanding prevalence and associated symptoms of vitamin deficiency and inadequacy will guide further research on this topic.

To ensure swine producers in Pennsylvania receive the most up-to-date and relevant information for their operations, it is essential to first identify and understand the reproductive and nutritional challenges observed across swine farms in the state. Differences in production strategies, herd health, and reproductive performance across farms of varying sizes must be considered to develop effective, size-specific nutritional and management recommendations. The substantial variability in herd size and management practices among Pennsylvania swine farms underscores the need for a thorough understanding of farm-specific challenges prior to offering practical guidance. This study aimed to determine the incidence of common health and reproductive challenges within swine herds in Pennsylvania. Additionally, serum vitamin A and D concentrations were assessed in a subset of these farms, providing insights into the vitamin status of sows in Pennsylvania.

## Materials and methods

### Ethics approval

All survey data was collected with Pennsylvania State University Internal Review Board’s approval (IRB: STUDY00024332). All animal procedures were approved by the Institutional Animal Care and Use Committee (IACUC: PROTO202402713) at The Pennsylvania State University.

### Survey distribution

Survey data was collected anonymously through Qualtrics (Qualtrics, Provo, UT) during the period of 1 May 2024, until 31 December 2024. Individuals were recruited for this survey through email, social media, and in-person events. Respondents were informed that the goal of this survey was “to gather information about reproductive challenges facing swine producers and investigate potential gaps in nutrition that may be related to these challenges”. All respondents had to confirm that they were over 18 years old, currently own pigs, consent to the use of their answers for evaluation, and that their farm was in Pennsylvania. Responses that did not meet those criteria or complete at least 95% of the survey were excluded.

### Questions asked

The survey questions asked are described in Table 1. Questions were divided into 3 categories. Questions in the first category aimed to assess herd size, number of gilts, sows, and boars, and the number of weaned pigs produced per year. Questions in the second category aimed to assess animal health issues, reproductive issues, feed sources, and management practices to determine how various farms in Pennsylvania maintain and manage their herds. Questions in the third category aimed to determine feeding, testing, and supplementation status, mortality rates at different stages of production, and the most common causes of mortality. The last question on the survey asked whether individuals were interested in continued participation in this research. Those who answered yes were taken to a separate survey where they filled out their name and contact information. Those individuals who answered yes were contacted to ask if they were interested in having feed and sow/gilt blood samples taken from their farm.

**Table 1.** Summary of questions asked in the survey.

Questions Asked	Options Available to Respondents
**At this time only Pennsylvania farms are eligible to take part in this survey, is your farm located in Pennsylvania?**	Yes, No
**What is your approximate average annual herd size?**	1–24, 25–49, 50–99, 100–199, 200–499, 500–999, 1000+
**How many breeding gilts do you have at your farm?**	Short answer response
**How many breeding sows do you have on your farm**	Short answer response
**How many breeding boars do you have on your farm?**	0–2, 3–5, 6–10, 11+
**How many weaned pigs does your farm produce in a year?**	0–24, 25–49, 50–99, 100–199, 200–499, 500–999, 1000+
**How many piglets per sow does your farm produce per year?**	Short answer response
**What kind of production efficiencies or management documentation do you record/track?**	Check all that apply. Breeding date, Conception/farrowing rate, Farrowing date, Litter performance (litter size, weight, born alive, stillborn, etc.), Wean dates, Mortality, Cross-foster movements, Medical records (vaccination dates, doses, antibiotic treatments, etc.), Other (write in)
**What are the sources for your pig feed?**	Check all that apply. Purchased complete feed, Home mixed ration, Home mixed ration with purchased concentrate &/or vitamin/mineral premix, Food waste NOT sourced from home on the same premises (i.e. plate waste, waste from industry or restaurant including meat, vegetables or milk waste products), Food waste from the home on the same premises (i.e. plate waste, waste from home) Other (write in)
**With respect to swine health what issues have you observed on your farm in the past 4 years?**	Check all that apply. Circo viral pathogens (PRRS, PED, Circovirus, etc.), Bacterial pathogens (brucellosis, leptospirosis, myoplasma, hyopneumonia, etc.), Intestinal stress, Respiratory distress, Transition stress (weaning, farrowing, etc.), Transport stress, Parasites (external and internal), High mortality, Thrift,/unthriftiness, Structural deformities—especially in piglets from sows reared on the farm, Humped back pigs, especially piglets from sows reared on the farm, Conjunctivitis, Lameness, Other (write in)
**What are your top reproductive challenges from the past 4 years?**	Rank your top three (3), with 1 being most important. Wean to estrus interval, Body condition, Nutrition, Low litter size, Pre-weaning piglet mortality, Sow mortality, Conception rate, Farrowing rate, Piglet birth weight, Abortions, Other
**Do you test your feed for vitamin and/or mineral status?**	Yes, vitamins, Yes, minerals, Yes, both, No, both,
**Do you supplement with any products for improved nutrition?**	Yes (short answer box to provide more information), No
**Do you regularly check body condition score?**	Yes, No
**Can you quantify your average mortality in sow herd?**	Please enter the average number of sows in a year. If willing, please share the most common reason(s) for sow mortality (short answer)
**Can you quantify your average preweaning piglet mortality percentage per year?**	0%–5%, 6%–10%, 11%–15%, 16%–25%, 25%+ If willing, please share the most common reason(s) for piglet mortality
**Can you quantify your average post weaning piglet mortality percentage per year?**	0%–5%, 6%–10%, 11%–15%, 16%–25%, 25%+ If willing, please share the most common reason(s) for piglet mortality

### On farm sample collections

All animal procedures were approved by the Institutional Animal Care and Use Committee (IACUC: PROTO202402713) at The Pennsylvania State University. Four respondents agreed to allow researchers to come out to their farm to obtain blood and feed samples from their herds. Blood was drawn from as many breeding females as possible, but no more than 14 were used per farm (total *n *= 28). Sows were restrained using a snare for sampling. Blood (≤5 mL) was collected by venipuncture of the jugular vein. Blood was collected using 10 mL vacutainer tubes without coagulant (Ideal Instruments, Canton, MA.). Once collected, blood tubes were placed on ice and protected from the light to prevent vitamin degradation. Samples were stored at 4°C until processing.

At the time of sample collection, researchers asked the producers about their sow’s history and production information. These questions included the breed, current body condition score, parity, and reproductive status of the sow. Producers were then asked to describe their sow’s reproductive history, including past reproductive performance, reproductive issues observed, and piglet survival rates, and other information they wanted to provide. Examples of additional information collected included the number of services prior to a successful breeding, information on litter performance such as replacement gilt/boar selection, and the reproductive performance of sows in her lineage.

### Serum processing

Blood samples were processed within 24 h of collection. Blood was centrifuged for 15 minutes at 1940 x g at 10°C, and serum was stored at -20°C protected from light until subjected to further analysis.

### Serum retinol analysis

Retinol was extracted from 100 µL of serum as previously described (Ross 1986). Extracted retinol was saponified using 5% (w/w) potassium hydroxide in 95% ethanol, extracted in hexane, and reconstituted in 100 µL of methanol with 150 pmol trimethylmethoxyphenyl-retinol (TMMP-ROH) as an internal standard. To analyze retinol concentrations, 10 µL of lipid extracts in methanol were injected onto a *Waters Acquity* ultra-performance liquid chromatograph with a BEH C18 column (Waters Acquity: inner diameter: 2.1 mm, length: 50 mm, particle size: 1.7 µm) coupled with a *Waters Acquity* photo diode array (PDA) detector (Waters Corporation; Milford, MA). A constant flow of 92.5% methanol, and 7.5% deionized water was used with a flow rate of 0.6 mL/minute and a run time of 1.2 minutes. Retinol concentrations were analyzed by a PDA detector using an absorbance wavelength of 325 nm with 4.8 nm resolution. Serum retinol concentrations were calculated based on the related area counts of the internal standard, TMMP-ROH.

### Serum 25(OH)D analysis

The concentration of 25-hydroxycholecalciferol plus 25-hydroxyergocalciferol (25(OH)D) in serum (*n *= 26) was quantified by enzyme-linked immunosorbent assay (ELISA) as per the manufacturer’s instructions using the Mouse/Rat 25-OH Vitamin D Elisa Kit (VID21-K01Eagle Bioscience Inc. Nashua, NH.). Sample absorbance was quantified using a spectrophotometer (SynergyH1, BioTek, Winooski, Vermont) at 450 nm. The supplied kit standards were utilized to generate a standard curve from 0 to 150 ng/mL and samples were diluted to ensure the concentrations were within the limits of the standard curve. The maximum detection limit was 145.9 µg/mL and the average coefficient of variation (CV) was 4.55%. Hemolyzed samples were found to affect the measurement of vitamin D in the serum, so all hemolyzed samples were excluded from further analysis (*n *= 9).

### Statistical analysis

Data collected from the survey was analyzed for descriptive statistics in Microsoft Excel (Redemont, WA, USA) and PC SAS (9.4; SAS, Cary, NC, USA). Data were analyzed using frequency statistics (Proc Freq) with a Fisher test for small counts. Pairwise analysis was used to evaluate differences between herd sizes. Serum vitamin A and D concentrations were analyzed using PC SAS. Serum data were tested for normality using Shapiro-Wilks test and assessed for outliers using Graph Pad Prism (GraphPad, Bostin, MA, USA). Retinol and 25(OH)D data were Log10 transformed to achieve a normal data distribution. Serum retinol data had a normal data distribution and were analyzed using one-way ANOVA and T-Tests. Serum 25(OH)D data did not have a normal distribution, and Kruskal-Wallis and Mann-Whitney U Test were utilized. Significance was declared at *P *≤ 0.05, and tendencies were declared at *P *≤ 0.10.

## Results

A total of 92 responses were collected from the survey. Of those, 47 were excluded for reasons including not meeting the completion threshold (95% complete), not being in Pennsylvania, or not consenting to the survey. The remaining responses (*n *= 45) were categorized by herd size, 1–49, 50–199, and 200+ as described in Table 2.

**Table 2.** Categorization of herd size by number of responses.

Herd Size	Number of Responses
**1–49**	34
**50–199**	7
**200+**	4

### Reproductive issues were different between herd sizes and selection frequency

**Fig. 1.**
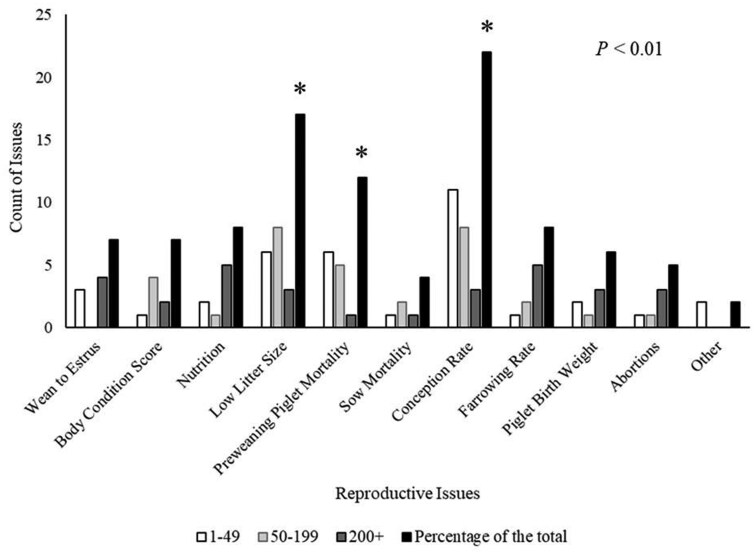
Reproductive issues reported on Pennsylvania swine farms. Selected issues indicated by a * are more important for respondents (*P *< 0.01). Statistics were calculated using a frequency test (proc freq).

Respondents were asked to select the three most important reproductive issues they have encountered over the past 4 years out of 11 options and rank them from 1 (most important) to 3 (third most important). Independent of rank, respondents report conception rate, low litter size, and preweaning piglet mortality more frequently than other options (*P *< 0.01, Fig. 1). Results are displayed as a count of respondents who experienced that reproductive issue, including issues by rank and total. Due to sample size limitations, reproductive issues could not be assessed by rank. Reproductive issues were also evaluated by frequency of selection of each herd size, and most issues were similar among herds. Preweaning piglet mortality (*P *< 0.05, Table 3) was numerically more important to respondents as herd size increased, although pairwise differences were not significant. Tendencies were observed when evaluating conception rate and low litter size (*P *< 0.10). Respondents with herds of 1–49 and 50–199 numerically reported a more significant issue with conception rate, and herds of 50–199 numerically reported low litter size being a significant issue in their operation. No pairwise evaluations of these issues were significant (*P *> 0.10). Respondents who selected “other” were asked what other issues they observed in their herd, responses included prolapses (*n *= 1) and silent estrus (*n *= 1).

**Table 3.** The percentage of reproductive issues observed among different herd sizes over the past 4 years, and the percentage of total respondents reporting each reproductive issue.

	Herd Size				
Reproductive Issues	1–49^1^	50–199^1^	200+^1^	*P*-value	Percentage of total respondents reporting this issue
**Wean to Estrus**	14.7	28.6	0	0.13	15.6
**Body Condition Score**	14.7	28.6	0	0.13	15.6
**Nutrition**	17.7	14.3	25	0.17	17.8
**Low Litter Size**	35.3	57.1	25	0.07	37.8
**Preweaning Piglet Mortality**	20.6	42.9	50	**0.04**	26.7
**Sow Mortality**	11.8	0	0	0.31	8.9
**Conception Rate**	50.0	57.1	25	0.08	48.9
**Farrowing Rate**	17.7	28.6	0	0.13	17.8
**Piglet Birth Weight**	14.7	14.3	0	0.24	13.3
**Abortions**	8.8	28.6	0	0.10	11.1
**Other**	2.9	0	25	0.14	4.4

Table 3 represents reproductive issue by herd size.

Bold text represents significant differences (*P *< 0.05).

1 Results are reported as a % of respondents per herd size who selected that reproductive issue.

Statistics were calculated using a frequency test (Proc Freq).

### Providing nutritional supplementation is associated with testing feed for vitamin/mineral status

Respondents who did not provide a nutritional supplement were less likely to test for vitamin or mineral status than those who provided nutritional supplements (*P *< 0.01. Table 4). Respondents could select from “Yes, both”, “Yes, vitamins”, “Yes, minerals”, and “No, both”. If respondents provided a nutritional supplement, they were asked what supplements they provided. Responses included several brands vitamin/mineral supplements or show feeds (*n *= 7), or specific ingredients such as additional l-lysine (*n *= 2), sunflower oil (*n *= 1), apple cider vinegar (*n *= 1), dried sea kelp (*n *= 1), walnuts (*n *= 1), and natural essential oils (*n *= 1).

**Table 4.** Percentage of respondents who test for vitamin/mineral status in their feed and if they provide a nutritional supplement.

	Nutritional Supplement	
Test for Vitamin/Mineral Status	No	Yes
**No, both**	55.8^a^	18.6
**Yes, both**	7.0	14.0^a^
**Yes, minerals**	2.3	2.3
**Yes, vitamins**	NA	NA

Table 4 represents whether respondents provide a nutritional supplement based on vitamin/mineral testing status.

NA = not applicable, no respondent selected this option.

a Represents pairwise differences (*P *< 0.05).

Results are reported as a percentage of respondents who selected each option.

A total of 4.44% of the data was removed because respondents did not answer either question.

Statistics were calculated using a frequency test (Proc Freq).

### Respondents utilized different feed sources depending on herd size

Respondents could select as many feed sources as they used on their farm, including complete purchased, home mixed, home mixed with a purchased vitamin/mineral premix, onsite food waste, offsite food waste, or other. Results are reported as a percentage of respondents for each herd size classification. Complete purchased feed was different (*P *< 0.05, Table 5) with herds of 1–49 and 200+ using it as a feed source more frequently than herds of 50–199. Herds of 50–199 tended to utilize home mixed rations with a vitamin/mineral premix more often than herds of 1–49 (*P *< 0.10). Herds of 1–49 tended to use onsite food waste more often than larger herds (*P *< 0.10). Respondents who selected “other” as an option were asked to provide more information regarding their feed source and they indicated hay (*n *= 2), vegetables (*n *= 2), alfalfa pellets (*n *= 1), and a diet containing no soy or corn (*n *= 1).

**Table 5.** The percentage of feed sources utilized by different herd sizes, and the percentage of total respondents using each feed source.

	Herd Size				
Feed Source	1–49^1^	50–199^1^	200+^1^	*P*-value	Percentage of total respondents reporting this feed source
**Complete Purchased**	73.5	42.9	75	**0.04**	68.9
**Home Mixed with Premix**	38.2	71.4	25	**0.03**	42.2
**Home Mixed**	5.9	0	25	0.15	6.7
**Onsite Food Waste**	26.8	0	0	0.06	20.0
**Offsite Food Waste**	5.9	0	0	0.57	4.4
**Other**	11.8	0	0	0.31	8.9

Table 5 represents feed source by herd size.

Bold text represents significant differences (*P *< 0.05).

Italicized numbers in the same row represent pairwise tendencies (*P *< 0.10).

1 Results are reported as a % of respondents per herd size who selected that feed source.

Results are reported as a percentage of respondents who selected that option per herd size.

Statistics were calculated using a frequency test (Proc Freq).

### Preweaning piglet mortality was affected by herd size

Herds of 1–49 report reduced preweaning piglet mortality than herds of 50–199 and 200+ (*P *< 0.01, Table 6). Additionally, herds of 50–199 report reduced preweaning piglet mortality compared to herds of 200+ (*P *< 0.05). Respondents were also asked what their most common cause of preweaning mortality was, and answers included crushing by sow (*n *= 16), stillborn (*n *= 6), low viability/runt piglets (*n *= 5), strep/illnesses (*n *= 3), deformities (*n *= 2), hypothermia (*n *= 1), scours (*n *= 1), sow aggression (*n *= 1), and unknown cause (*n *= 2). Post-weaning mortality was reported as 0%–5% by most responders (*n *= 39) except one respondent who reported post-weaning mortality rates of 6%–10% (*n *= 1).

**Table 6.** The percentage of preweaning piglet mortality as a percentage of respondents from each herd size.

Preweaning Piglet Mortality	Herd Size		
	1–49^1^ ^,^ ^2^	50–199^1^	200+^1^ ^,^ ^2^
**0%–5%**	64.71	42.86	0
**6%–10%**	20.59	14.29	25
**11%–15%**	2.94	14.29	0
**16%–25%**	2.94	28.57	25

Table 6 represents the percent preweaning piglet mortality as a percentage of respondents by farm size.

Significant difference of *P *< 0.01 observed.

Pairwise differences observed between herd size 1–49 and 50–199 and 200+ (*P *< 0.01).

Pairwise difference observed between herd size 50–199 and 200+ (*P *< 0.05).

1 Results are reported as a % of respondents per herd size who observed that preweaning piglet mortality.

2 Herd sizes 1–49 and 200+ had respondents not answer this question. Herd size 1–49 had 8.82% of farms did not respond, and herd size 200+ had 50% of farms did not respond.

Statistics were calculated using a frequency test (Proc Freq).

### Frequency of health issues reported varied by herd size

The percentage of herds who reported issues with viral pathogens, bacterial pathogens, respiratory distress, and intestinal stress were different when compared by herd size (*P *< 0.05, Table 7). Herds of 200+ tended to report more issues with viral pathogens than herds of 1–49 (*P *< 0.10). Although not statistically significant, numerically issues with bacterial pathogens, respiratory distress, and intestinal stress appeared to increase as herd size increased. Issues with parasites were not reported in herds of 200+ and were only reported as an issue in herds of 1–49 and 50–199. “Other” issues were only reported in herds of 200+. Tendencies were observed in lameness, structural deformities, and humpbacks (*P *< 0.10), with frequency of the issue being reported increasing as herd size increases. Respondents were asked if they observed any other health issues, responses included rotavirus type A and C (*n *= 1), hemorrhagic bowel syndrome (*n *= 1), and porcine sapovirus (*n *= 1).

**Table 7.** The percentage of health issues observed among different herd sizes over the past 4 years, and the percentage of total respondents reporting each health issue.

	Herd Size				
Health Issues	1–49^1^	50–199^1^	200+^1^	*P*-value	Percentage of total respondents reporting this issue
**Viral Pathogens**	8.8	28.6	50.0	**0.02**	15.6
**Bacterial Pathogens**	11.8	14.3	50.0	**0.04**	15.6
**Respiratory Distress**	20.6	42.9	50.0	**0.04**	26.7
**Parasites**	32.4	57.1	0	**0.03**	33.3
**Transport Stress**	14.7	28.6	25.0	0.11	17.8
**Transition Stress**	14.7	28.6	25.0	0.11	17.8
**Intestinal Stress**	5.9	28.6	25.0	**0.04**	11.1
**High Mortality**	8.8	14.3	25.0	0.14	11.1
**Thrift**	8.8	14.3	25.0	0.14	11.1
**Lameness**	6.7	28.6	25.0	0.06	13.3
**Structural deformities**	2.9	14.3	25.0	0.07	6.7
**Humpback**	2.9	14.3	25.0	0.07	6.7
**Conjunctivitis**	2.9	0	0	0.75	2.2
**Other**	0	0	25.0	**< 0.01**	4.4

Table 7 displays health issues observed over the past 4 years by the percentage of herd size.

Bold text represents significant differences (*P *< 0.05).

Italicized numbers in the same row represent pairwise tendencies (*P *< 0.10).

1 Results are reported as a % of respondents per herd size who selected that health issue.

Statistics were calculated using a frequency test (Proc Freq).

### Record keeping strategies varied depending on herd size

In this survey, respondents are more likely to prioritize different records based on herd size. Veterinary records were more likely to be maintained in herds of 1–49 and 50–199 than herds with 200+ pigs (*P *< 0.05, Table 8). The likelihood of respondents recording conception/farrowing rate and breeding date records was greater in herds of 50–199 compared to herds of 1–49 and 200+ (*P *< 0.05). The likelihood of respondents recording litter performance varied depending on herd size (*P *< 0.05), with herds of 50–199 tending to record this more often than herds of 1–49 (*P *< 0.10). The incidence of respondents recording mortality (*P *< 0.01) was higher among herds of 50–199 than herds of 1–49 (*P *< 0.05). A tendency was observed in cross-foster movements (*P *< 0.10), with larger herds more likely to report that they recorded cross-fostering than smaller herds.

**Table 8.** List of possible records respondents could utilize and the frequency as a percentage of respondents in each herd size to maintain those records, and the percentage of total respondents utilizing each record.

	Herd Size				
Records	1–49^1^	50–199^1^	200+^1^	*P*-value	Percentage of total respondents utilizing records
**Veterinary Records**	88.2	85.7	50	**0.04**	84.4
**Conception/Farrowing Rate**	55.9	85.7	50	**0.05**	60
**Breeding Date**	79.4	100	50	**0.03**	80
**Farrowing Date**	76.5	85.71	75	0.16	77.8
**Litter Performance**	64.7	100	50	**0.02**	68.9
**Wean Date**	61.8	71.4	50	0.11	62.2
**Mortality**	52.9^a^	100^b^	75^a^b	**<0.01**	62.2
**Cross-Foster Movements**	11.8	28.6	25	0.08	15.6
**Other**	2.2	14.3	50	**< 0.01**	8.9

Table 8 represents records utilized based on herd size.

Numbers are reported as a percentage of farms who utilized records based on herd size.

Bold text represents significant differences (*P *< 0.05).

Italicized numbers in the same row represent pairwise tendencies (*P *< 0.10).

a Different superscripts (a, b) denote differences within row (*P *< 0.05).

1 Results are reported as a % of respondents per herd size who maintain that record.

Statistics were calculated using a frequency test (Proc Freq).

### Serum retinol

**Fig. 2.**
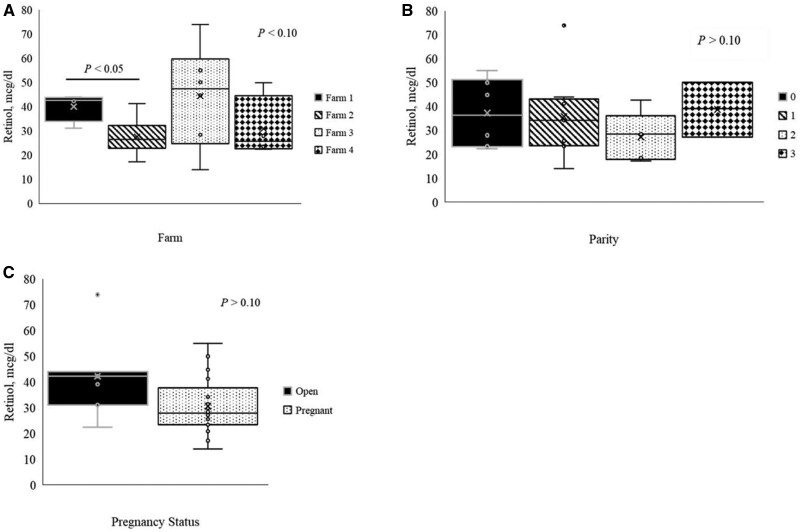
Serum retinol concentrations from sampling on Pennsylvania swine farms. a) Serum retinol concentrations on the four farms sampled. b) Parity did not affect serum retinol concentrations. c) Pregnancy status did not affect serum retinol concentrations.

An overall effect was observed when serum retinol concentration was evaluated by farm (*P *< 0.10). Sows from farm 1 had greater serum retinol concentrations (*P *< 0.05, Fig. 2a) than sows from Farm 2. While no other differences were observed by farm, farms 1 and 3 numerically had greater serum retinol than farms 2 and 4. Sampled sows were parities 0 (*n* = 6), 1 (*n* = 9), 2 (*n* = 4), 3 (*n* = 3), 5 (2), 6 (*n* = 2), and 8 (*n* = 1). Parities 5, 6, and 8 were excluded from analysis due to restricted sample size. Serum retinol was not affected by parity (*P *> 0.05. Fig. 2b). Sows were categorized as pregnant or open for evaluation of pregnancy status. Pregnancy status had no effect on serum retinol concentration (*P *> 0.05, Fig. 2c).

### Serum 25(OH)D

Sows from farm 2 had greater serum 25(OH)D concentrations (627.2 ± 191.8 ng/mL, *n* = 13) compared to sows from farm 3 (69.7 ± 21.7 ng/mL, *n* = 3. *P *= 0.01). Farms 1 and 4 were excluded from this analysis due to restricted sample size. Serum 25(OH)D was not affected by parity [*P* > 0.05, parity 1 (497.3 ± 257.9 ng/mL, *n* = 5), parity 2 (548.0 ± 304.9 ng/mL, *n* = 4), parity 3 (88.5 ± 28.8 ng/mL, (*n* = 3)]. Sows from parity 0, 5, 6, and 8 were excluded from analysis due to restricted sample size. The effect of pregnancy status on serum 25(OH)D concentrations could not be evaluated due to restricted sample size.

## Discussion

Pennsylvania is ranked 12^th^ in the United States of America in pork production and hosts a diverse population of farms of different herd sizes, management techniques, and reproductive issues. Despite this diversity, these differences have not been systematically quantified, yet it is well established that variations in housing systems, feeding strategies, and overall management can influence reproductive outcomes and animal health. In this study we performed a comprehensive assessment of producer observations of health and reproductive issues, preweaning piglet mortality, record keeping, the use of vitamin/mineral testing, and nutritional supplementation in Pennsylvania.

When evaluating differences in health concerns, reproductive challenges, piglet mortality, and record keeping practices, herd size consistently influenced producer responses. Among reproductive issues, conception rate, low litter size, and preweaning piglet mortality were the most frequently selected, while the other challenges were reported at relatively consistent frequencies. Interestingly, while conception rate was chosen as the most important reproductive issue, only 60% of respondents selected it as an option for what records they kept on herd performance, highlighting a disconnect in reported importance and reported record-keeping practices among respondents. Similarly, preweaning piglet mortality rates were reported as a major challenge among herds of less than 50 pigs, but only approximately half of those respondents maintained mortality records. Comparatively, respondents with herds of over 50 pigs reported higher preweaning piglet mortality rates. A relationship between herd size and preweaning mortality has previously been suggested, with small herd size being associated with an elevated risk of preweaning mortality. It could be speculated that the differences in preweaning mortality and health issues between small and large herds may be due to herds of over 50 sows more frequently maintaining mortality records, having improved management of sows and piglets during the preweaning phase, and generally having more advanced facilities with more labor available (Hoshino and Koketsu 2009; Koketsu et al. 2020).

Several health issues that were affected by herd size, with herds of 1–49 numerically having less observed health issues than the larger herd sizes. Viral and bacterial pathogens and respiratory distress were numerically reported as an issue more often as herd size increased. Previous reports have suggested that large herd sizes have an increased risk of influenza A, pleuritis, pneumonia, and porcine respiratory and reproductive syndrome virus (Gardner et al. 2002; Evans et al. 2008; Fablet et al. 2016; Takemae et al. 2016), highlighting the need for appropriate biosecurity precautions and management strategies to prevent disease spread, particularly in large operations. Reported issues with parasites, however, were not a problem in herds of 200+, and were only reported as an issue in herds of 1–49 and 50–199. This is most likely due to housing style, with smaller herds usually being kept outdoors rather than in a full confinement operation. However, a limitation of the study was that housing style was not included as a question so we cannot confirm the method of housing different respondents use. Importantly, differences in farmer knowledge, attentiveness, and management intensity are likely between small- and large-scale operations. Larger farms, which often represent a major source of income, are more likely to employ additional labor, which may partly explain differences in how farmers perceive the health status of their herds. While differences were observed when evaluating health issues reported by herd size, all issues except respiratory distress and parasites were reported by less than 20% of respondents, indicating that most herds are not experiencing widespread illness. A difference in vitamin/mineral testing and nutritional supplementation was also observed, with respondents who do not test for vitamin/mineral status being less likely to provide a nutritional supplement compared to respondents who do test for vitamin/mineral status. This difference in testing and supplementation could be from lack of available resources or understanding. It has been suggested that the current NRC recommendations represent the minimum vitamin requirements (Yücel and Taşkin 2018), with many nutritionists supplementing vitamins and trace minerals well above the NRC requirements (Faccin et al. 2023). There was significant variation between nutritionists surrounding the supplementation levels utilized for all vitamins and trace minerals, but particularly for fat-soluble vitamins such as vitamins A and D (Faccin et al. 2023). It is important for producers, especially those formulating their own rations at home, to recognize that current recommendations may not fully meet the modern sow’s nutritional needs for optimal productivity. Conversely, over-supplementation can be costly, making it essential to provide only the amount required to achieve peak performance. Sows and piglets who are deficient in vitamin A are more likely to see increased mortality piglet mortality (Brief and Chew 1985), decreased embryonic implantation (Yin et al. 2021), and reduced immune functions (Vlasova et al. 2013). Sows given supplemental vitamin D produced piglets with improved muscle (Hines et al. 2013) and skeletal development (Zhou et al. 2023). Diets sufficient in vitamin requirements are critical to maintaining a healthy herd and productive animals.

While differences in production strategies, health and reproductive issues, and preweaning piglet mortality were observed between herds of different sizes, we believe our results were impacted by the limited number of participants who had herds of 200+. However, as 91% of swine farms have fewer than 2,000 animals, collectively accounting for 12% of the state’s pig population (Cook and Schulz 2023), the results of this survey reflect the husbandry practices on most of the swine production operations in Pennsylvania. No statistically significant pairwise observations could be made when evaluating that herd size due to this limitation, and this can only be solved by increased participation.

Several serum samples were hemolyzed and removed from analysis, which affected our ability to report results for 25(OH)D by parity and pregnancy status. Hemolyzed samples can alter the absorbance reported when evaluating results, so those samples were excluded. It has previously been observed that stage of gestation effects serum 25(OH)D3 concentrations (Stenhouse et al. 2022), but we could not evaluate serum 25(OH)D by pregnancy status due to limited sample size. To date there have been few reports of serum retinol and 25(OH)D in the modern hyperprolific sow. Although serum samples were only tested from a small number of sows on a limited number of farms in Pennsylvania, these findings provide insights into the serum concentrations of retinol and vitamin D in the modern sow and highlight a need for education on the importance of appropriate vitamin supplementation to maximize productivity and animal health, while minimizing the costs of supplementation. Further investigation using a large sample size at different stages of production will be able to comprehensively assess whether there has been a change in the vitamin requirements of the modern sow and generate educational materials to support swine producers in performing the best supplementation strategies.

## Conclusion

This study quantified the diversity of health and reproductive issues, preweaning piglet mortality, record-keeping, vitamin/mineral testing, and nutritional supplementation among pig producers in Pennsylvania, and highlighted the corresponding need for tailored education and support. While serum retinol and 25(OH)D values from select farms provide additional information of the nutritional status of the modern sow, these preliminary findings highlighted the need for further research and education surrounding vitamin requirements and supplementation practices in sows. Although the results presented in this study are from Pennsylvania swine farms, the diversity of farm size and management practices utilized in Pennsylvania are reflective of those observed throughout the United States of America, highlighting the need for tailored education and support based on farm size.

## Data Availability

The datasets used and/or analyzed during the current study are available from the corresponding author on reasonable request.
